# Custom-made Subperiosteal Implants: A Finite Element Analysis on Monoblock and Dual Implant Systems in Atrophic Maxilla

**DOI:** 10.7150/ijms.89411

**Published:** 2023-10-16

**Authors:** Mustafa Ayhan, Abdulkadir Burak Cankaya

**Affiliations:** Istanbul University Faculty of Dentistry Department of Oral and Maxillofacial Surgery, Istanbul, Turkey

**Keywords:** Custom Made Implant, Subperiosteal Implant, Severe Bone Resorption

## Abstract

This study aims to investigate and compare the stress distribution, displacement, and bone loading of monoblock and dual custom-made subperiosteal implant systems in atrophic maxilla using finite element analysis (FEA). A total of 11 patients with insufficient bone tissue for conventional implant treatment were included in the study. Customized subperiosteal implant designs were generated using the 3D average models obtained from patients' computed tomography (CT) scans. Two different models were produced: a monoblock that covered the entire maxillary bone and a dual implant system where two mirror-imaged implants covered the left and right halves of the maxillary bone separately. We have calculated residual stress values formed on the implant models and jaw bone models separately. In addition, the highest displacement values formed on the implants and the highest stress values formed on abutment parts have also been observed in this study.

Results showed that the stresses formed on implants that are under the mastication forces were significantly lower than the yield strength of the selected material, indicating that plastic deformation would not occur under static load. The dual implant geometry demonstrated a substantial reduction in stress compared to the monoblock structure. The highest von Mises stress values for the monoblock implants ranged from 131 MPa to 206 MPa, while those for the dual structure ranged from 124 MPa to 178 MPa. The highest residual stress values on the upper jawbone were observed in the M6 implant model, and the lowest was seen in the M1 and M3 models at 12 MPa. Displacement values under static load showed that loads on the implant would be below 0.21 mm.

In conclusion, custom subperiosteal implants are a viable treatment option for patients with insufficient bone tissue for conventional implants. Dual implant systems were found to have lower stress and displacement values compared to monoblock structures, indicating a potential advantage in clinical use. However, mono implants may have benefits in cases of immediate teeth loading due to their ability to absorb and distribute occlusal forces better.

## Introduction

Dental implants have become a widely accepted treatment modality for patients with missing teeth due to their high success rates and ability to restore function and aesthetics 1. However, patients with atrophic maxilla often present with insufficient bone volume, making the placement of conventional implants challenging 2. Several techniques, such as autogenous bone grafting or iliac bone grafting techniques, may be painful and require longer treatment periods. Zygoma implants have been suggested as a treatment alternative to avoid such procedures that include bone augmentation. However, this method has various problems, including sinus infections, eye-related complications, and prosthesis attachment points at undesirable points 3-5.

Dahl first introduced subperiosteal implants in the 1940s. In those years, to produce an implant compatible with supporting the bone, it was first necessary to take measurements from inside the mouth. Then, the implant design was made on the model created from the measurement, and the implants were produced as casting from Vitallium and Tantalum. The implants produced later were placed into the mouth with a second surgery. However, over time, subperiosteal implants were abandoned due to serious compatibility problems, implants being exposed to the mouth, and stability problems due to not using screws for fixation 6.

However, today, the use of digital technology in dentistry has resulted in revolutionary results in terms of the adaptation of implants to the patient's mouth, their design, and applicability.

Subperiosteal implants are a less invasive alternative to conventional implants, designed to be placed on the bone surface and covered by the periosteum rather than being inserted into the alveolar bone 7. These implants are custom-made based on the patient's anatomy and have shown promising results in terms of function and success rates 8. However, limited information is available regarding the biomechanics and stress distribution of subperiosteal implants, especially in the atrophic maxilla 9.

Understanding the biomechanics and stress distribution of subperiosteal implants in the atrophic maxilla is crucial for optimizing implant design and achieving long-term clinical success. This study's results could help clinicians select the most suitable subperiosteal implant design for their patients, improving patient outcomes and satisfaction.

Finite element analysis (FEA) is a valuable tool in investigating dental implants' biomechanics and stress distribution in different clinical scenarios 10. It provides valuable insights into the performance of various implant designs and materials, allowing for the optimization of implant geometry and minimizing complications associated with mechanical loading 11. Only a few studies have focused on the biomechanical analysis of subperiosteal implants using FEA 9,12.

This study aims to investigate and compare the stress distribution and displacement of customized monoblock and dual subperiosteal implant systems in atrophic maxillas using FEA. The influence of implant design, thickness, and screw diameter on stress and displacement values will be examined to provide a better understanding of the biomechanics of these implant systems. We believe that this study will contribute to the existing knowledge on the biomechanics of customized subperiosteal implants and provide valuable information for clinicians working with patients presenting with atrophic maxilla. It is anticipated that the findings will aid in the optimization of implant design and material selection, ultimately improving the success rates and long-term outcomes of subperiosteal implant treatments.

## Materials and Methods

### Patient and Data Selection

Between 2018 and 2021, 49 patients who applied to our clinic for implant treatment but found insufficient bone tissue for conventional implant treatment in clinical and radiographic examinations were examined for custom subperiosteal implant treatment. In further investigations, 33 patients with uncontrolled comorbid factors, bisphosphonate use, cleft lip and palate history, or smoking were evaluated as unfavorable regarding a subperiosteal implant. All eligible patients are over 60 years old. Despite the indication, four patients refused to be treated voluntarily. Subperiosteal implant treatment was applied to 12 admitted patients at different times. Pre-op and post-op radiographic information of 11 patients who underwent the application was transferred to digital media. One patient was not included in the study because post-op follow-ups could not be performed for reasons unrelated to the study.

### Numerical Data Processing

Volumetric binary files (VBF) of CT scans of 11 patients were grouped as one file cluster. The model-to-model distance module of 3DSlicer (open source) was performed on the file cluster, and a distance map between 11 models was computed. This distance map creates corresponding point-to-point distance tables with anatomically selected points. Using a principal-component analysis module with computation of the mean group decided, a mean value is determined for the group. After the mean value determination for the group, this data is used to generate a template model with the Shape Variation Analyzer module of the 3DSlicer (Open Source). The Shape Population module visualizes the developed model, and the resulting 3D model is used for all the following subperiosteal implant designs. This re-generated 3D model is based on all the mean values of the 11 patients' data and contains all anatomically relevant points.

### Construction of Geometric Models

Models in stereolithography (STL) format designed with BioTechnica medical engineering company were imported into CAD (Computer Aided Design) (Rhinoceros 4.0; 3670 Woodland Park Ave N, Seattle, WA 98103 USA) software.

**Figure 1:**
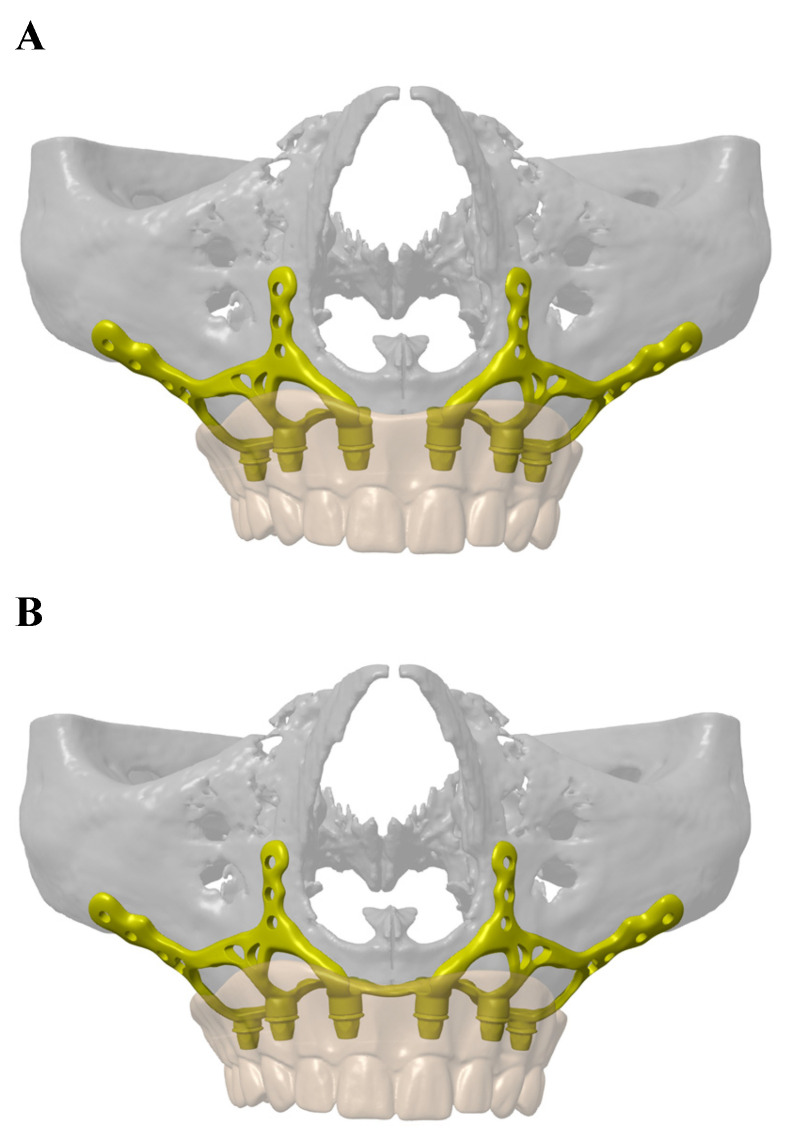
** A:** Dual implant system in which two mirror-imaged implants cover the left and right half of the maxillary bone separately. **B:** Monoblock implant system that covers all of the maxillary bone as one piece

Two different models have been produced: a dual implant system in which two mirror-imaged implants cover the left and right half of the maxillary bone separately and a monoblock that covers all of the maxillary bone as one piece (Figure 1/A-B).

Each implant type is designed with different material thicknesses (T) (1mm and 1,5mm) with different screw diameters (SD) (1,5mm and 2mm). Model types and mash sizes are given in Table 1.

The reverse engineering module of the CAD software was used to convert the 3D models taken as point clouds into solid models. The 3D solid model required to analyze the implant geometry was obtained. Minimizing the deviation between the accepted 3D model and the point cloud data is imperative. For this, deviation analysis has been made for all surfaces obtained by region definitions. The amount of deviation was determined as 0.05 mm.

**Figure 2:**
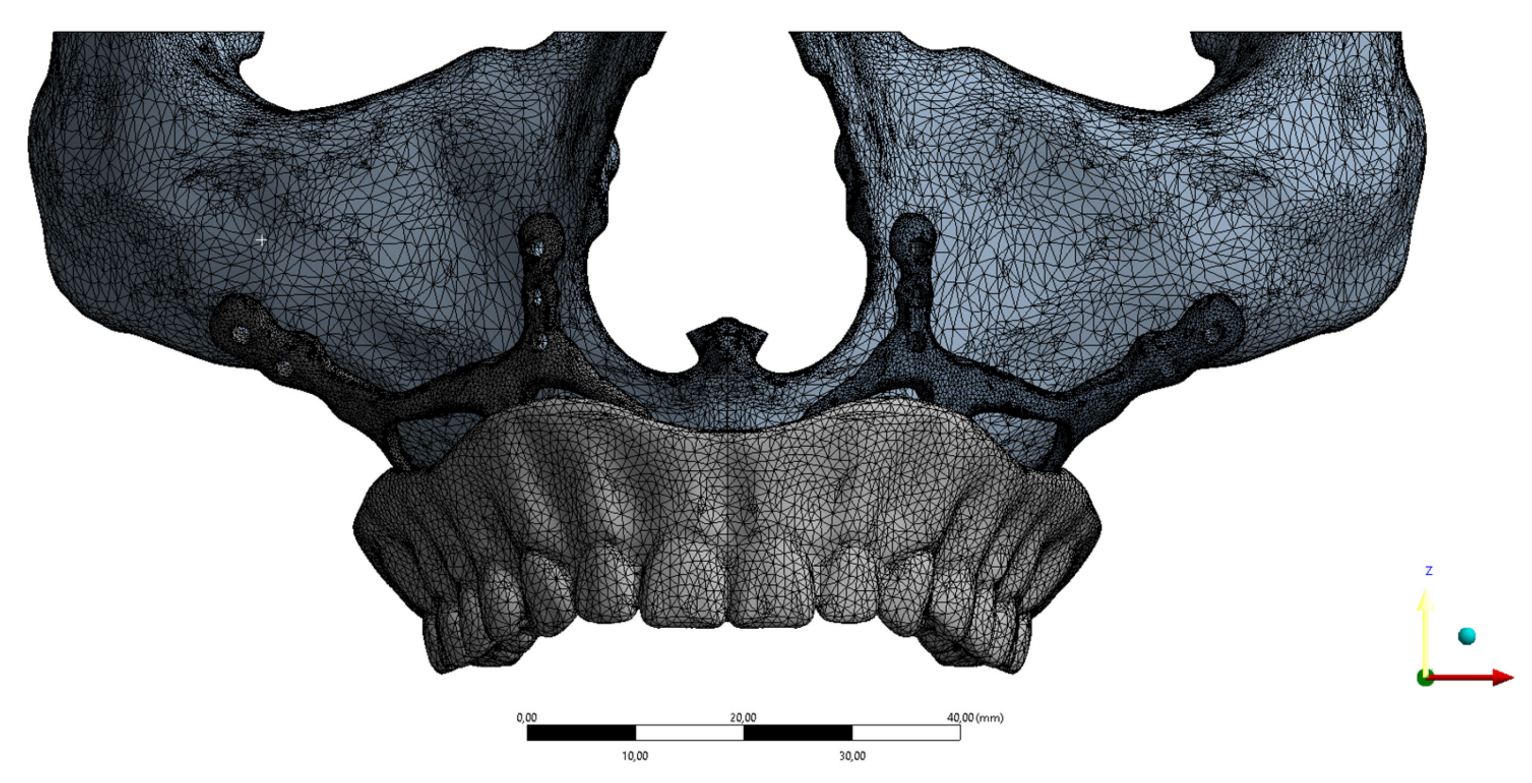
Implant mesh model image.

FEA was used to determine the stress distribution, on the whole, consisting of bone and implant. The 3D solid model obtained with CAD was transferred to FEA (Abaqus/ CAE 2017), and a three-dimensional mesh consisting of 1033174 - 116801 nodes and 589462-716185 elements with VRMesh Studio (VirtualGrid Inc, Bellevue City, WA, USA). The finite element method is a numerical method that allows us to obtain information about the structure by dividing the form into a finite number of small elements and solving a finite number of equations instead of an infinite number. For this reason, the established solution network is vital for the calculation result. An adaptive mesh was applied in the finite element model that was established. The mesh sizes used in the parts forming the whole, the modulus of elasticity, and the poison ratios of the materials used are given in Figure 2. The solution matrix is calculated as a tetrahedron mesh type and parabolic element. The mesh size was calculated as 0.5mm.

### Load Conditions and Stress Analysis

**Figure 3:**
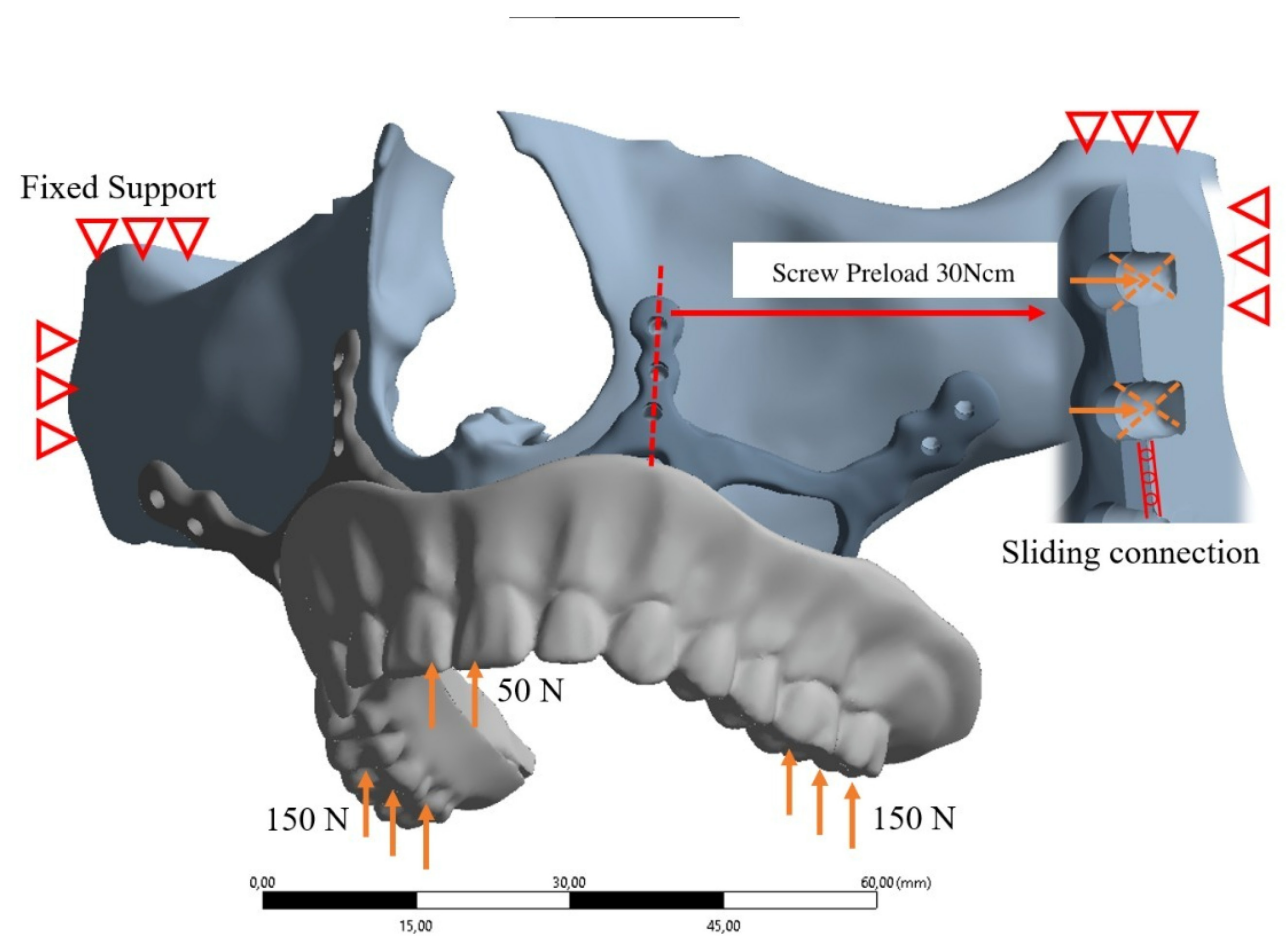
Boundary conditions used in FEA solutions

In our study, stress distribution and analysis were performed for eight different models under a vertical load of 150 Newton on both posterior sides and 50 Newton load on the anterior region vertically as described in Figure 3. The stress distribution and effects on the implants were calculated with the Von-Misses yield criterion. Since the bone is not in a homogeneous structure and von Misses can only be used in homogeneous systems, the stress analysis on the bone was calculated according to the Piola-Kirchhoff stress tensors theorems. Although implant and bone stress values were calculated separately, stress measurements were made based on bone-implant contact points to obtain meaningful results. In addition, the highest displacement values formed on the implants and the highest stress values formed on abutment parts have also been observed in this study.

Boundary conditions used in FEA solutions are given in Figure 3. The maxillary region was limited to 6 degrees of freedom from four regions. The implant and the maxillary bone connection are fixed with a 7-point screw connection. A virtual screw connection was used in the connections, and the preload of the screws was determined as 30Nm. An axial load of 50N was applied to the anterior teeth and 150N to the posterior teeth to simulate the mastication load on the dental veneer.

In our study, the reliability coefficient was determined as 2 to evaluate the plastic deformation to be detected in the material. According to this safety coefficient, the von-misses stress value on the implant should be less than half the yield strength of the material to avoid plastic deformation. The yield strength of the Ti6Al4V material is 897 MPa and values below 447 MPa, and it is assumed that it does not undergo plastic deformation. Ti6Al4V material is considered fully plastic until 114MPa and can return to its original form without permanent deformations. These values are significantly different from the maxilla bone 13 . The yield strength of the maximal bone material is 104 MPa, and with values below 23 MPa, it is assumed that it does not undergo plastic deformation. Material properties for the test are given in Table 2.

### Study Type and Location

This experimental laboratory study was carried out in Istanbul University Faculty of Dentistry, Department of Oral and Maxillofacial Surgery (Turkey) with the partnership of BioTecnica Engineering, Medical Company (Turkey). İstanbul University Local Ethics Committee issues ethical approval of the study with the number of 2023/12 Rev-1.

## Results

It has been observed that the stresses formed on implants under chewing load are much lower than the yield strength of the selected material and the dual implant geometry has a significant contribution to the reduction of stresses. In the mono structure implants, the observed von Mises stress values are at the highest level of 206 MPa in the M5 model and the lowest level of 131 MPa in the M2 model, while in the dual structure, the highest level is 178 MPa in the M7 model, and the lowest is 124 MPa in the M4 model (Table 3).

**Figure 4:**
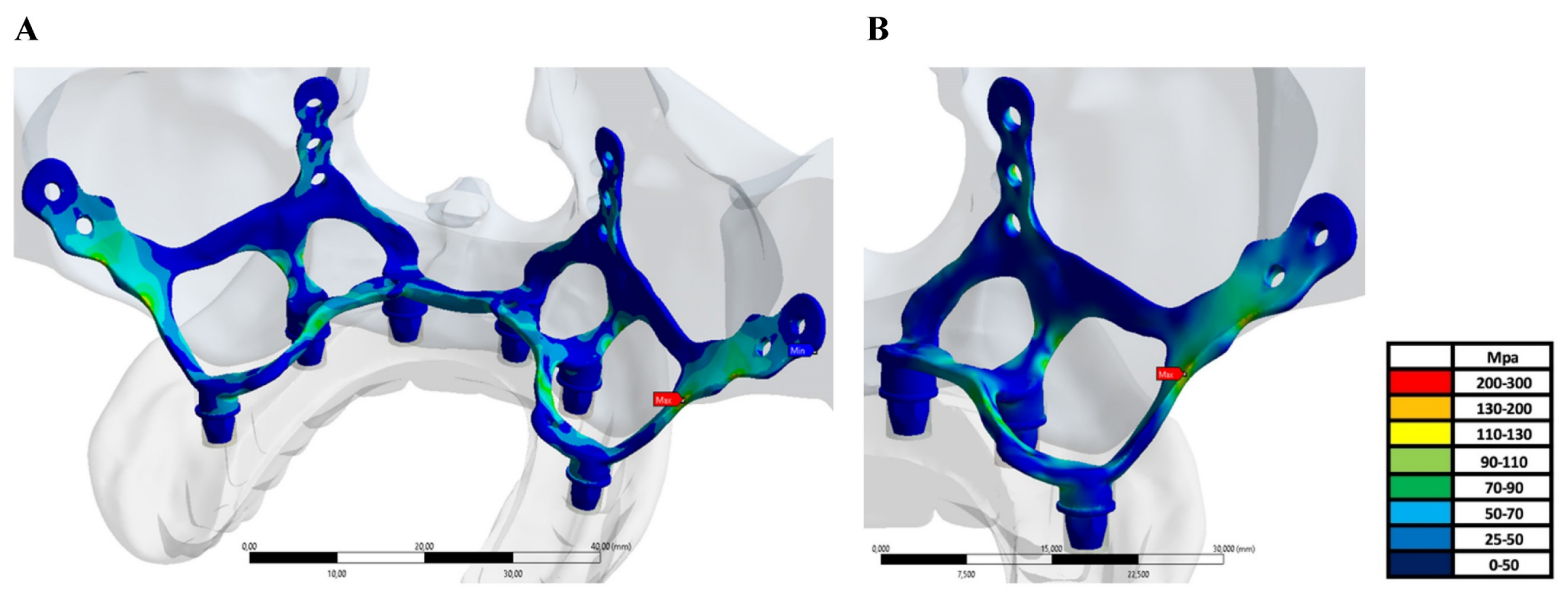
** A-B:** The highest von Mises stress values formed on the implant.

On the other hand, when the highest stress values formed on the implant are examined regionally, it is seen that they occur in the region where the implant extends to the zygomatic area for all implants (Figure 4a-b).

When the residual stress values formed on the upper jaw are examined, the highest residual stress value occurs in the upper jaw area where the M6 implant model is used, while the lowest upper jaw residual stresses are seen in the M1 and M3 models at 12 Mpa (Table 4). Unlike von Mises stresses, it has been observed that thin-walled mono-structured implants provide a homogeneous load distribution on the stresses on the upper jaw and thus cause lower residual stresses to form. On the other hand, using large screws and thick implants increases the stresses on the upper jaw.

Table 5 shows the displacement values under static load formed on the implants. When axial and total displacement values are examined separately, it is seen that the loads formed on the implant will be below 0.21 mm. The highest displacement values are observed in the posterior regions. Displacements occur at a higher rate in mono structures compared to dual structures. Additionally, 1 mm thick implants show more displacement than 1.5 mm thick ones. However, the differences between the groups are negligible. The fact that some displacement is observed in the implants indicates that the stress forces formed on them can be transferred as displacement. In other words, the formed stresses are absorbed to some extent due to the implant's displacement capability. It is thought that as the thickness of the implants increases, the displacement value will decrease, and von Mises stresses will be greater. Therefore, it is not considered appropriate to target near-zero displacement values. Finally, in the design verification analyses performed assuming a safety factor of 2, it has been determined that the implants are in a safe area.

The von Mises stresses formed on the abutment parts where the prosthesis is placed are evaluated in Table 6. With the M2 model in the mono structure using a 1.5 mm screw, a stress of 15.7 MPa was obtained, while in the M4 model with a dual structure, a stress of 18.1 MPa occurred on the abutment (Table 6). It is evaluated that mono-structured implants will contribute to lower bending stresses compared to dual structures. More significant effects of using screws with different diameters are observed. When the values formed are examined, it has been determined that it is a negligible stress value compared to the yield strength of the material used.

## Discussion

Subperiosteal implants are usually preferred over conventional dental implants because they provide a viable treatment option for patients with significant bone atrophy, who might otherwise be unable to receive dental implants. Conventional dental implants require sufficient healthy bone for successful osseointegration and long-term stability 14. However, patients with severe bone loss or resorption, often caused by long-term edentulism or systemic conditions such as osteoporosis, may not have enough bone volume for the placement of traditional implants 15. In such cases, subperiosteal implants offer an alternative solution, as they can be custom-designed to fit the patient's existing bone anatomy and do not rely on osseointegration for their stability 16.

Another advantage of subperiosteal implants is that they may eliminate the need for bone grafting procedures, which can be invasive, time-consuming, and costly 17. Bone grafting is often necessary to build up the bone volume for the placement of conventional implants, but it can involve significant patient morbidity and an extended treatment timeline 18. In contrast, subperiosteal implants are designed to be supported by the patient's remaining bone and soft tissue, circumventing the need for grafting and shortening the overall treatment duration 19. Thus, subperiosteal implants may be preferred in certain clinical situations where conventional dental implants are not feasible due to inadequate bone volume or when patients want to avoid bone grafting procedures.

This preliminary study examined stress distribution in dual and mono-design subperiosteal implants under physiological forces. Our findings represent an initial exploration of the potential benefits of these implant designs, with direct comparisons to existing literature being limited at this stage. We discovered significant differences in von Mises stress between the designs. Mono implants, featuring a thinner structure encompassing the entire maxillary bone, provide a more uniform load distribution on the upper jaw, reducing residual bone stress. This characteristic may contribute to better clinical outcomes and long-term success. Conversely, dual implants, with their thinner structure covering the left and right maxillary bone halves independently, enable improved load distribution and stress absorption.

Mono implants exhibited the highest von Mises stress value at 206 MPa (M5 model) and the lowest at 131 MPa (M2 model), whereas dual implants demonstrated a range of 178 MPa (M7 model) to 124 MPa (M4 model). The distinct stress distribution can be attributed to dual implants covering the left and right maxillary bone halves separately, which may enhance load distribution and stress absorption. As a result, the lower stress levels in dual implants could potentially minimize implant failure or bone resorption risks, potentially improving clinical outcomes.

Additionally, this study investigates the impact of implant design on bone loadings during chewing forces. Notably, results indicate that mono implants exert less force on the bone compared to dual implants, despite contrasting von Mises stress values. For example, the M1 and M3 models exhibited the lowest residual stresses on the upper jaw at 12 MPa. This suggests that mono implants may have a more favorable influence on surrounding bone tissue, despite dual implants displaying lower stress values within their structure, as observed in the M4 model at 124 MPa.

Our results imply that mono implants may be more suitable for immediate teeth loading scenarios due to their enhanced force absorption and distribution capabilities. Mono implants yield a more homogeneous load distribution on the upper jaw, reducing residual stress on the bone. This feature is particularly advantageous in immediate loading cases, where increased stress concentration could result in complications such as implant failure or bone resorption. Moreover, this study revealed that mono implants generally exhibit higher displacement values than dual implants. Counterintuitively, this displacement under load may prove beneficial in immediate loading situations by effectively dispersing occlusal forces and promoting implant stability. One notable advantage of the thin structure of mono implants is the potential reduction in gingival recession risk during chewing forces. As the implant structure is thinner, it exerts less pressure on the surrounding gingival tissue during functional loads, such as chewing 20.

Despite mono implants' potential benefits in stress distribution and immediate loading, surgical challenges associated with their placement should be considered. Mono implants entail more complex and demanding placement than dual implants due to the single-piece structure, requiring precise positioning and alignment for proper fitting and optimal load distribution. Surgeons need advanced technical skills and experience, as well as thorough preoperative planning for successful outcomes.

It is important to note that, according to our results, screw use appears to be a more critical variable affecting bone stress than implant design. This finding underlines the need for clinicians to pay close attention to screw selection and placement during implant surgery. It is thought that one of the reasons for complication is the metal thickness of the implants 20. To optimize outcomes, future research should investigate the optimal combination of implant design, thickness, and screw use, taking into account both the stress distribution on the implant and the bone loading. This could lead to the development of more effective subperiosteal implant designs and improved patient outcomes.

In conclusion, while mono implants provide certain advantages, their surgical complexity warrants careful consideration by clinicians. The potential benefits should be weighed against surgical challenges, ensuring the necessary expertise and resources are available for optimal results.

## Conclusions

1. Dual implants exhibited lower von Mises stress within the implant structure than mono implants. However, mono implants applied less force on the bone, providing a more homogeneous load distribution on the upper jaw and resulting in lower residual stresses forming on the bone.

2. Screw use appeared to be a more critical variable affecting bone stress than implant design. Therefore, carefully considering screw size and placement is crucial during implant surgery.

3. Mono implants may have advantages in cases of immediate teeth loading due to their ability to better absorb and distribute occlusal forces. Their higher displacement values under load could be beneficial for reducing stress concentration on the bone-implant interface and enhancing implant stability.

## Figures and Tables

**Table 1:** Mesh sizes and material thicknesses of the groups.

Models	Mesh Size	Number of Node	Number of Elements
**M1** - T:1.0mm, SD:1.5 Mono Impant	0.5mm	1041506	594216
**M2** - T:1.5mm, SD:1.5 Mono Impact	0.5mm	1052027	600219
**M3** - T:1.0mm, SD:1.5 Dual Implant	0.5mm	1080443	709023
**M4** - T:1.5mm, SD:1.5 Dual Implant	0.5mm	1187300	716185
**M5** - T:1.0mm, SD:2.0 Mono Impant	0.5mm	1033174	589462
**M6** - T:1.5mm, SD: 2.0 Mono Impant	0.5mm	1043610	592415
**M7** - T:1.0mm, SD: 2.0 Dual Implant	0.5mm	1071799	701289
**M8** - T:1.5mm, SD: 2.0 Dual Implant	0.5mm	1164801	709355

**Table 2:** Mechanical properties of the test subjects.

Material	Tensile Strength(MPa)	Yield Strength (MPa)	Elasticity(GPa)	PoissonRatio	%Displacement
Ti-6Al-4V (ISO58323:2021)	965	897	114	0.33	12
Maxilla Bone	283	104	23	0.32	1.2

**Table 3:**
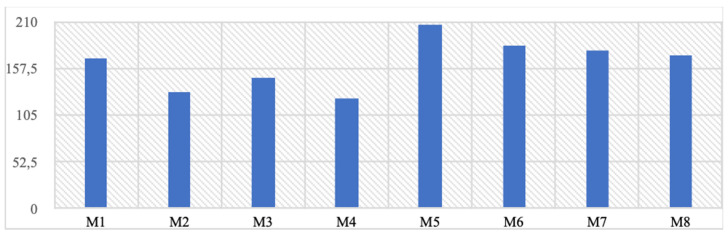
von Mises and residual stress values formed on the implant, y-axis von Mises stress (Mpa).

**Table 4:**
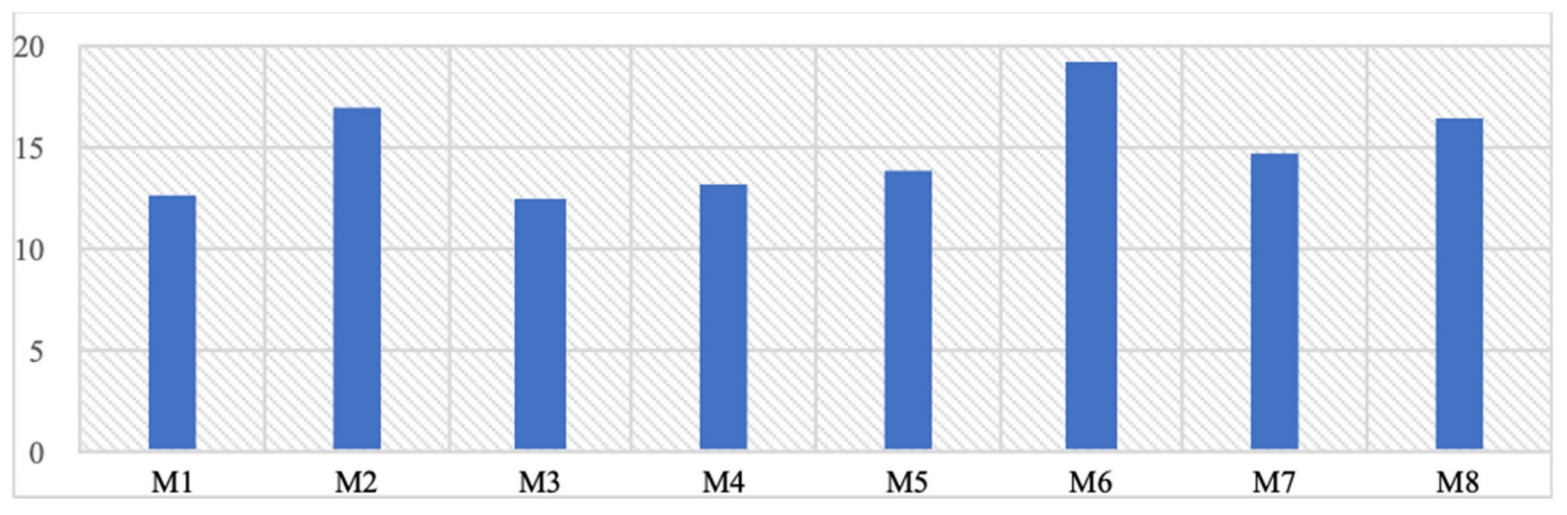
The highest residual stress values formed on the upper jawbone (Mpa).

**Table 5:**
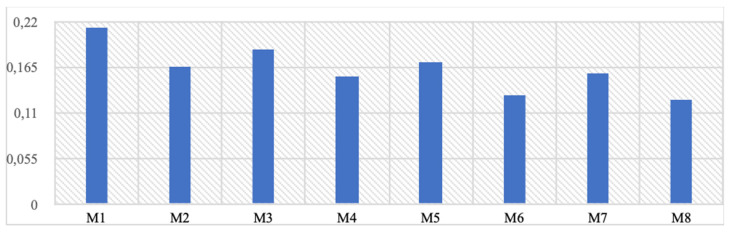
The highest displacement values formed on the implant (Mpa).

**Table 6:**
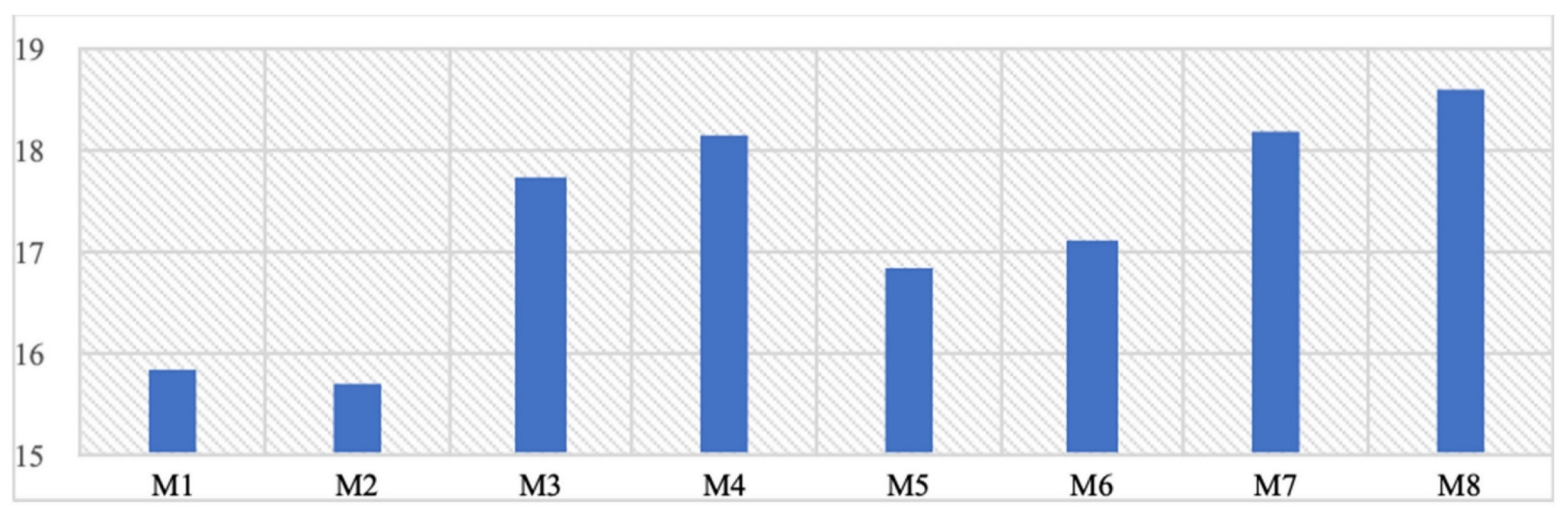
The highest stress values formed on abutment parts (Mpa).
